# Cannabidiol (CBD) differentially shapes systemic cytokines and transcriptomic profiles in the liver and spleen: insights from a male mouse model

**DOI:** 10.1007/s00438-026-02525-w

**Published:** 2026-09-28

**Authors:** Jakub Żurowski, E. Ocłoń, I. Jasielczuk, T. Szmatoła, K. Mizera-Szpilka, E. Semik-Gurgul, S. Sawicki, Artur Gurgul

**Affiliations:** 1 Department of Basic Sciences, Faculty of Veterinary Medicine, University of Agriculture in Kraków, Redzina 1C, 30-248 Krakow, Poland; 2 Laboratory of Recombinant Proteins, Faculty of Veterinary Medicine, University of Agriculture in Kraków, Redzina 1C, 30-248 Krakow, Poland; 3 Department of Infectious Diseases and Public Health, Faculty of Veterinary Medicine, University of Agriculture in Kraków, Redzina 1C, 30-248 Krakow, Poland; 4 Department of Animal Molecular Biology, National Research Institute of Animal Production, Krakowska 1, 32-083 Balice, Poland; 5 Department of Animal Reproduction, Anatomy and Genomics, University of Agriculture in Krakow, Mickiewicza 24/28, 30-059 Krakow, Poland

**Keywords:** Cannabidiol, Cytokines, transcriptomics, Immunometabolism, Liver, Spleen

## Abstract

Cannabidiol (CBD) is widely studied for its anti-inflammatory and immunomodulatory properties, yet its relationship with systemic cytokine responses and organ-level transcriptional changes remains incompletely defined. Here, we investigated the dose- and time-dependent effects of CBD on circulating pro-inflammatory cytokines and the transcriptomic profiles of the liver and spleen in male C57BL/6J mice. Animals received CBD intraperitoneally at 0.2, 10, or 20 mg/kg body weight for either 2 days (short-term, ST) or 28 days (long-term, LT), with vehicle-treated controls. Serum IL-1β, IL-6, and TNF-α were measured by ELISA, and RNA-Seq was performed on liver and spleen to characterize differential gene expression and enriched biological processes. CBD significantly reduced IL-1β and TNF-α after ST treatment across doses, whereas IL-6 decreased primarily during LT exposure, with the strongest reduction at day 14 and partial persistence to day 28 at higher doses. Transcriptomic responses were markedly organ- and regimen-dependent. The liver transcriptome exhibited pronounced, dose- and time-dependent remodeling, with the largest number of differentially expressed genes at 20 mg/kg in ST and broad changes across doses in LT. The gene enrichment analyses indicated modulation of mitochondrial and translational pathways after ST high-dose exposure, lipid and fatty acid metabolism during LT, as well as circadian-associated regulation at higher-dose LT treatment, with innate immune–associated signaling terms enriched at the highest dose. In contrast, the spleen displayed comparatively modest transcriptional changes, with limited differential expression in LT and a clearer response only after ST exposure to 20 mg/kg, where altered genes showed trends related to lipid-associated processes. Together, these findings demonstrate that CBD attenuates circulating pro-inflammatory cytokines while inducing strong, dose- and time-dependent hepatic transcriptional reprogramming and relatively limited splenic transcriptomic shifts. The integration of systemic and organ-specific data supports a model in which CBD acts as a systemic immunometabolic modulator rather than a simple immunosuppressant.

**Supplementary Information:**

The online version contains supplementary material available at https://doi.org/10.1007/s00438-026-02525-w.

## Introduction

Cannabidiol (CBD) is a non-psychoactive phytocannabinoid obtained from the *Cannabis sativa* plant that has raised considerable attention in recent years for its potential therapeutic properties. Unlike Δ9-tetrahydrocannabinol (THC), CBD does not produce intoxicating effects, making it a potential candidate for clinical and preclinical studies (Martinez Naya et al. 2024). In addition to its action in the central nervous system, numerous studies report evidence that CBD exhibits a range of interesting properties, such as being anti-inflammatory, immunomodulatory and antioxidant (Mujahid et al. 2025). These can be possibly associated with multiple mechanisms, in particular related to the endocannabinoid system (involving CB1 and CB2 receptors), peroxisome proliferator-activated receptor gamma (PPARγ), and modulation of intracellular signaling pathways such as NF-κB and MAPK (Martinez Naya et al. 2023). These pathways are also critical for controlling inflammatory gene expression, cytokine production, and immune cell function, which makes CBD a potent agent in immune system modulation.

The immune system maintains homeostasis and protects the organism from infections and tissue damage resulting from the infectious agent’s activity. Pro-inflammatory cytokines, such as interleukin-1 beta (IL-1β), interleukin-6 (IL-6), and tumour necrosis factor alpha (TNF-α), are key mediators of immune responses (Schuerwegh et al. 2003). To be precise, IL-1β is an important mediator of the inflammatory response and is involved in a variety of cellular activities, including cell proliferation, differentiation, and apoptosis (Lopez-Castejon and Brough 2011), IL-6 regulates acute-phase responses and B cell differentiation (Tanaka et al. 2014), and TNF-α orchestrates systemic inflammatory signaling (Grabarek et al. 2017). Dysregulation of these cytokine secretion mechanisms contributes to chronic inflammation, autoimmune disorders, and even tissue injury (Kim and Moudgil 2008; Noster et al. 2016).

In recent reports by various authors, CBD was found to modulate cytokine production (Sermet et al. 2021), suppress excessive inflammation (Xiong et al. 2012), and influence immune cell proliferation and apoptosis (Rieder et al. 2010), yet the detailed molecular mechanisms remain incompletely recognised. Several body organs are implicated in immune function maintenance and regulation. The organs, such as the spleen and liver, seem to play complementary and crucial roles in these processes (Brummer et al. 2025). The spleen functions as a central lymphoid organ, which is designed for blood filtering and detecting pathogens. It coordinates both innate and adaptive immune responses and contains various immune cell populations, including T and B lymphocytes, macrophages, dendritic cells, and NK cells, which produce cytokines and mediate systemic immune responses (Bronte and Pittet 2013; Lewis et al. 2019). As for the liver, apart from its metabolic and detoxification roles (also in CBD clearance), it is an immunologically active organ, containing, e.g. Kupffer cells, liver sinusoidal endothelial cells, and other immune cell populations. These cells contribute to immune surveillance, clearance of pathogens and toxins, and modulation of systemic inflammation (Robinson et al. 2016; Parlar et al. 2023). Changes in both those organs’ gene expression can reflect both local and systemic responses to bioactive compounds (such as CBD), including modulation of immune cell proliferation and activity, cytokine signalling, metabolic pathways activity and oxidative stress responses.

Transcriptomic analysis of these organs should allow for an assessment of molecular changes induced by CBD and to complement systemic cytokine measurements. In the previous studies, it was shown that CBD can influence gene expression related to inflammatory signalling, lipid metabolism, and immune cell activation in various tissues (Yang et al. 2019; Mujahid et al. 2025). Despite this, the integration of global transcriptomic changes in both spleen and liver with cytokine profiles is still severely lacking. Therefore, the present study aimed to investigate the immunomodulatory effects of CBD in mice by measuring key pro-inflammatory cytokines (IL-1β, IL-6, TNF-α) and performing transcriptomic analyses of the liver and spleen. We hypothesised that CBD administration would modulate immune responses by altering cytokine production and gene expression patterns associated with inflammation, immune regulation, and metabolic pathways, providing mechanistic insights into its potential therapeutic effects.

## Methods

### Animals, CBD treatment and tissue sampling

In brief, male C57BL/6J mice aged 60 days were used in the study. Animals were housed in groups of six in standard polycarbonate cages (37 × 21 × 15 cm) in a dedicated animal facility compliant with Polish and EU animal welfare regulations. The rooms were maintained at 22 ± 2 °C and 55 ± 10% humidity under a 12 h light/12 h dark cycle with 15 air changes per hour. Mice had ad libitum access to water and a normocaloric diet, and were provided with environmental enrichment. Animal health and welfare were monitored daily or every three days, depending on the experimental group.

Mice were randomly assigned to short-term (ST; 2 days, *n* = 24) or long-term (LT; 28 days; *n* = 24) cannabidiol (CBD) treatment groups. The rationale for the ST and LT treatments is to assess the potentially different effects of sporadic (acute) and chronic CBD use. Within each group (*n* = 6), animals received CBD at doses of 0.2, 10, or 20 mg/kg body weight, or vehicle only (control). Cannabidiol (> 99% purity, NMID512B, MERCK, USA) was dissolved in saline containing 2% Tween 80 and administered once daily (200 µL) via intraperitoneal injection. Animals were weighed weekly, and CBD concentrations were recalculated accordingly. In the long-term group, mean body weight increased from approximately 25.1 g to 28.9 g over the 28-day period, resulting in a corresponding adjustment of stock concentrations to maintain accurate mg/kg dosing throughout the study. All experimental procedures were approved by the II Local Ethics Committee in Kraków (approval no. 90/2022).

After completion of CBD administration (at least 35 min after the final dose following a 12 h fast), mice were euthanized by isoflurane inhalation followed by cervical dislocation. The liver and spleen were rapidly excised and collected into 1.5 mL tubes containing 200 µL of stayRNA™ reagent (A&A Biotechnology, Poland) to preserve RNA integrity by inhibiting RNase activity. Following overnight incubation in stayRNA™, the reagent was removed according to the manufacturer’s instructions, and tissue samples were stored at − 20 °C until RNA extraction.

### Blood sampling and cytokine analysis

For the determination of IL-1β, IL-6 and TNF-α concentrations, blood samples were collected at three defined stages of the experiment: (A) at the end of ST treatment, (B) on day 14 of LT treatment, and (C) at the end of LT treatment. The day 14 time point was added to assess changes in cytokine levels over the course of LT exposure. Blood was collected from the tail vein during the experiment (approximately 60 µL per animal), whereas terminal samples were obtained immediately after euthanasia and transferred into K3-EDTA tubes. In cases where the collected volume was insufficient for analysis, samples from animals within the same experimental group were combined to obtain the required volume, while ensuring a minimum of three replicates per group. Following collection, blood samples were centrifuged (2,000 × g for 10 min at 4 °C), and the resulting plasma was separated and stored at − 20 °C until analysis. Cytokine concentrations were measured using commercially available ELISA kits (Thermo Fisher Scientific) according to the manufacturers’ protocols, with absorbance determined at 450 nm using TECAN Infinite M200 PRO microplate reader. The intra-assay and inter-assay coefficients of variation were 7.2% and 9.8% for TNF-α, 5.8% and 6.7% for IL-6, and 5.1% and 5.7% for IL-1β, respectively.

### RNA purification and RNA-Seq library preparation

Frozen tissues were thawed on ice, and total RNA was extracted from all 48 samples using the AllPrep DNA/RNA Qiagen kit following the manufacturer’s instructions. RNA quality was assessed with the TapeStation 4150 (Agilent Technologies, USA) and quantified using the Qubit system (Thermo Fisher Scientific, USA). High-quality RNA (RIN > 8) was used (200 ng per sample) to construct directional mRNA libraries with the CORALL mRNA-Seq V2 kit (Lexogen, Austria) for liver, and QuantSeq 3’ mRNA-Seq V2 Library Prep Kit with UDI (Lexogen, Austria) for spleen. Libraries were sequenced commercially on an Illumina NovaSeq 6000 system (2 × 150 bp). Raw sequencing data are available in GEO (GSE261716) and SRA (BioProject PRJNA1088484) for liver, and GEO (GSE328789) and SRA (BioProject PRJNA1456538) for spleen.

### RNA-Seq data analysis

Sequencing reads underwent quality control with FastQC (v0.11.9) and were trimmed using Flexbar (v3.5.0)(Dodt et al. 2012) to remove adapters, low-quality bases, and short reads (also the second read for spleen). Cleaned reads were aligned to the mouse reference genome GRCm39 using STAR (v2.7.5c)(Dobin et al. 2013) and counted with Htseq-count (v1.99.2) (Anders et al. 2015). Differential expression, normalization, clustering, and principal components analysis (PCA) were performed with DESeq2(Love et al. 2014) via the iDEP2.0(Ge et al. 2018) platform. PCA was used to explore expression variability. Enrichment analyses for Gene Ontology (GO) biological processes were conducted iDEP2.0. Genes or pathways with an FDR-adjusted p-value < 0.05 (Benjamini-Hochberg correction (Benjamini and Hochberg 1995) were considered significant; however, for genes, a fold change criterion of > 2 was also applied.

### qPCR validation for RNA-Seq

For RNA-Seq validation, three genes showing differential expression in at least two comparisons were selected, including upregulated (*Nr1d1*,* Ciart*, in single treatments) and downregulated (*Fam222a* in both ST and LT treatments) and three other altered genes (*Ypel2*,* Them7*,* Ighg2b*). cDNA was synthesised from 300 ng total RNA using the High-Capacity cDNA Reverse Transcription Kit (Thermo Fisher Scientific) for half of the analysed liver samples (three per group, *n* = 24). RT-qPCR was conducted with AmpliQ 5× HOT EvaGreen^®^ qPCR Mix Plus (ROX) (Novazym, Poland) and primers spanning two adjacent exons (Supplementary File 7). Each sample was run in triplicate on a QuantStudio™ 7 Flex system. Relative expression was calculated using the ΔΔCt method (Pfaffl 2001), with *Ppia* as a reference gene as suggested in Tatsumi et al. (2008).

### Statistical analysis

Cytokine data were analysed using JASP statistical software. Data distribution was first assessed with the Shapiro-Wilk test. Normally distributed data were evaluated with ANOVA followed by Tukey’s post-hoc test. Correlation between RNA-Seq and RT-qPCR results was assessed in JASP statistical software and the Pearson correlation coefficient.

## Results

### Changes in plasma cytokine concentrations following CBD treatment

Plasma concentrations of IL-1β, IL-6, and TNF-α were assessed after short-term (ST; 2 days) and long-term (LT; 14 and 28 days) CBD administration.

After 2 days of ST treatment, IL-1β levels were significantly reduced across all CBD doses (*p* < 0.001), decreasing from 69.68 ± 2.41 pg/ml in controls to 57.06 ± 5.26, 52.36 ± 2.14, and 49.04 ± 1.14 pg/ml in the 0.2, 10, and 20 mg/kg groups, respectively, corresponding to an approximate 18–30% reduction. A similar pattern was observed for TNF-α, with concentrations declining from 107.91 ± 2.08 pg/ml in controls to 83.58 ± 5.89, 66.65 ± 0.68, and 59.12 ± 5.81 pg/ml, representing a ~ 20–45% decrease across increasing doses (*p* < 0.001). In contrast, IL-6 levels were not significantly altered following ST treatment.

At day 14 of LT treatment, IL-1β and TNF-α levels remained comparable to controls, indicating no sustained effect at this stage. In contrast, IL-6 concentrations were significantly reduced at all tested doses (*p* < 0.01), decreasing from 67.79 ± 2.15 pg/ml in controls to 49.88 ± 1.07, 54.62 ± 3.34, and 56.81 ± 3.10 pg/ml, corresponding to a ~ 16–26% reduction, with the strongest effect observed at the lowest dose.

After 28 days of treatment, IL-1β levels did not differ significantly between groups. However, IL-6 concentrations remained markedly reduced at higher doses, reaching 28.37 ± 1.67 and 26.70 ± 2.09 pg/ml at 10 and 20 mg/kg, respectively, compared with 45.27 ± 3.79 pg/ml in controls (*p* < 0.001), representing an approximate 40–45% decrease, while the lowest dose showed no significant effect. At this time point, TNF-α levels were also significantly reduced across all CBD-treated groups (*p* < 0.01), decreasing from 72.96 ± 2.90 pg/ml in controls to 64.13 ± 1.05, 64.42 ± 2.18, and 64.61 ± 1.58 pg/ml, corresponding to a ~ 12% reduction.

**Fig. 1:**
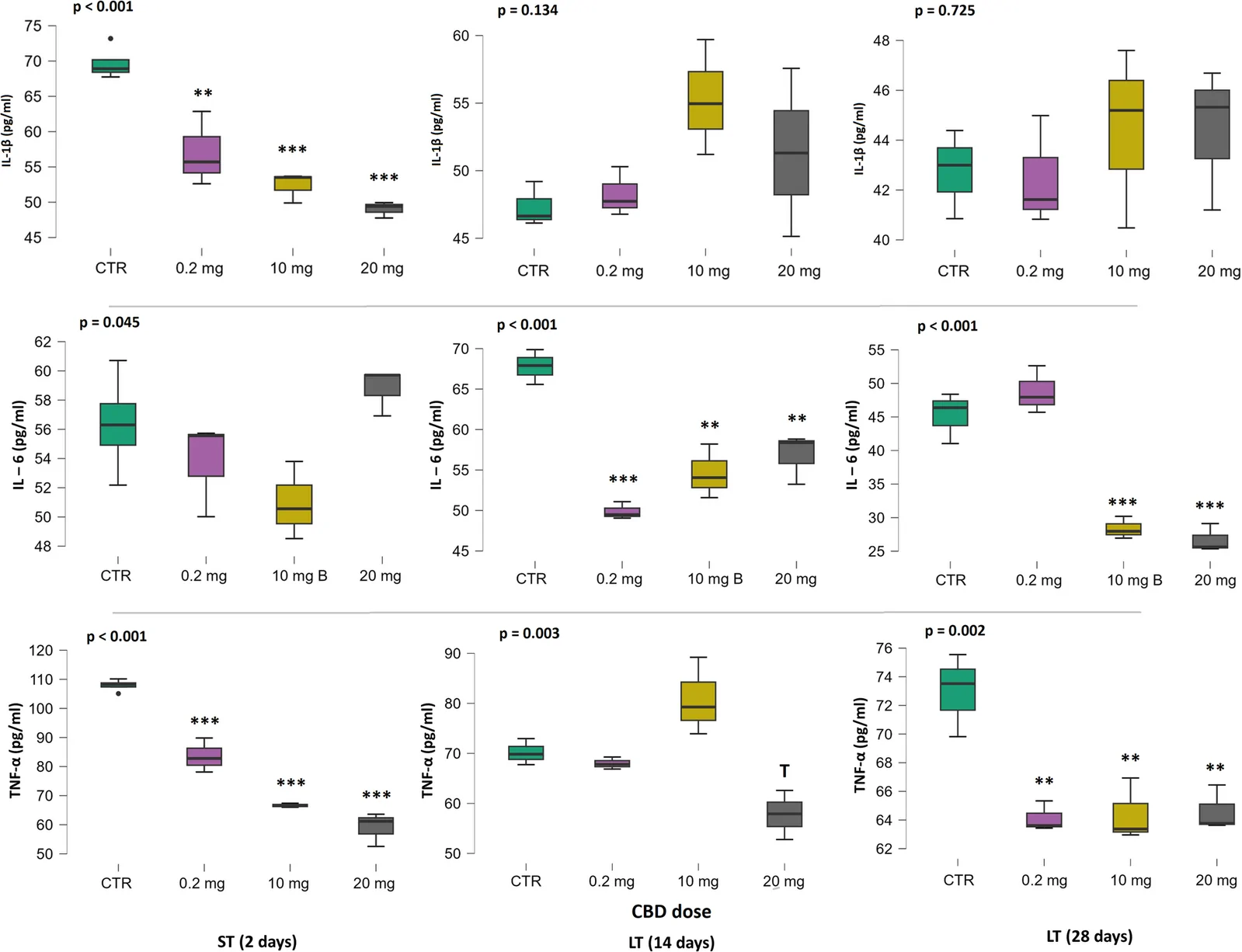
Changes in serum cytokine concentrations (IL-1β, IL-6, TNF-α) following different CBD treatments. ST – short term (2 days); LT – long term (28 days). *p* < 0.05; *p < 0.01; **p < 0.001. p-values are shown for comparisons with the control group; the global ANOVA p-value is also provided

Overall, CBD induced cytokine-specific and time-dependent effects, characterized by early reductions in IL-1β and TNF-α, and a delayed but sustained decrease in IL-6 during prolonged treatment (Fig. 1, Supplementary File 1).

### Changes in the transcriptome of the liver following CBD treatment

For liver samples, an average of 32.4 million (SD = 7.5) raw reads were generated, of which 93.9% (SD = 1.3) passed initial filtering. Of the filtered reads, an average of 74.7% (SD = 3.75) were uniquely mapped to the reference genome. Most of these reads were assigned to genes (75.9%; 19.9 million on average) (Supplementary File 2).

**Fig. 2:**
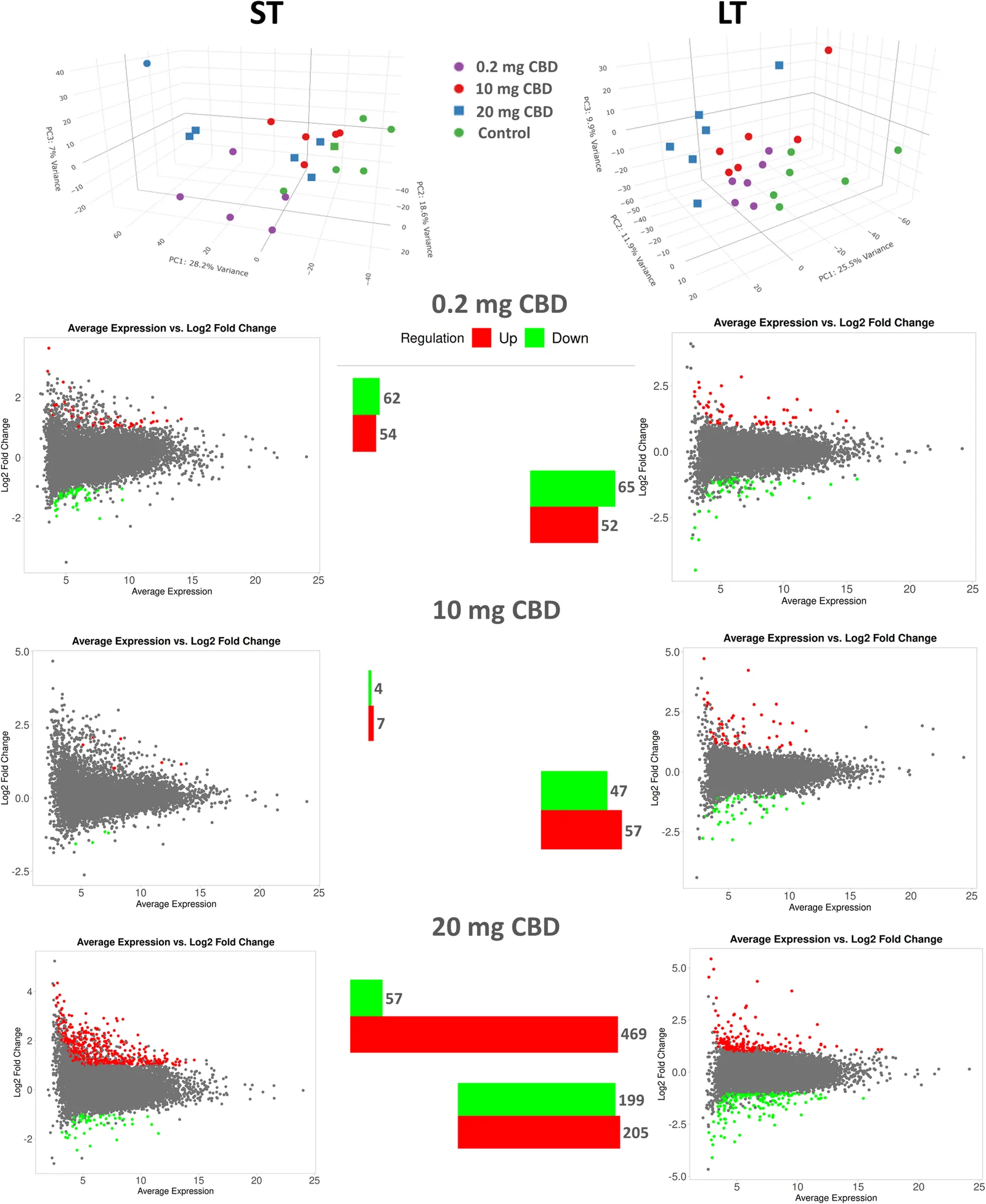
Changes in the hepatic transcriptional profile following CBD administration for two (ST) or 28 (LT) days. The figure presents PCA results for the first three principal components and MA plots illustrating gene expression changes induced by individual CBD doses. The number of differentially expressed genes identified for each treatment is also shown

Initial evaluation of the transcriptomic profiles in the ST group using PCA revealed that one sample from the control group and one from the 0.2 mg/kg b.w. CBD-treated group strongly deviated from the remaining samples within their respective groups and were therefore excluded from further analyses. The remaining samples clustered largely according to treatment group, with some overlap; however, a clear dose-dependent shift in expression profiles was observed. The greatest divergence from the control group in transcriptomic profiles was detected for samples treated with the highest applied CBD dose (Fig. 2). In the LT treatment, PCA revealed a clear separation between the control and treated samples based on their expression profiles, with the greatest divergence observed in samples from the highest CBD dose group. Additionally, some within-group heterogeneity in expression profiles was also detected, with the highest variance observed in the control and the 10 mg/kg CBD-treated groups (Fig. 2).

Differential expression analysis of separate genes revealed that CBD altered the expression of large number of genes, but this effect varied among applied doses. The lowest CBD dose affected 116 genes (54 upregulated and 62 downregulated). The intermediate dose, affected only 11 genes, while the highest CBD dose affected 526 genes, of which 469 (89.1%) were upregulated (Fig. 2) Of the genes 2 (*Fam222a*,* Nr1d1*) were commonly affected in all treatments and another 24 were common for two of the treatments (Supplementary File 3). In LT treatment, CBD affected, depending on the dose, form 104 (10 mg CBD) to 404 (20 mg CBD) genes, of which a comparable amount was up- and downregulated (Fig. 2). 14 genes were common for all LT treatments. What is more, one gene was commonly affected for all ST and LT treatments (*Fam222a*) (Supplementary File 3).

The overrepresentation analysis performed for genes altered by ST treatments revealed that the 0.2 mg/kg CBD dose led to downregulation of genes associated (FDR < 0.05) with GO Biological Process (BP) categories related to steroid and mineralocorticoid hormone biosynthesis, secretion, and metabolism (with a strong emphasis on aldosterone and corticosteroid pathways), regulation of endocrine and systemic processes, developmental and morphogenetic processes (notably tissue, epithelial, skin, and organ morphogenesis), cell differentiation and adhesion, and metabolic and transport processes (particularly lipid metabolism/export and amino acid transport). Together, these categories indicate coordinated regulation of hormonal signaling, development and tissue remodeling, and cellular metabolic homeostasis. Genes upregulated by the lowest CBD dose did not show any significant overrepresentation of GO BP categories (Supplementary File 4, Fig. 3).

**Fig. 3:**
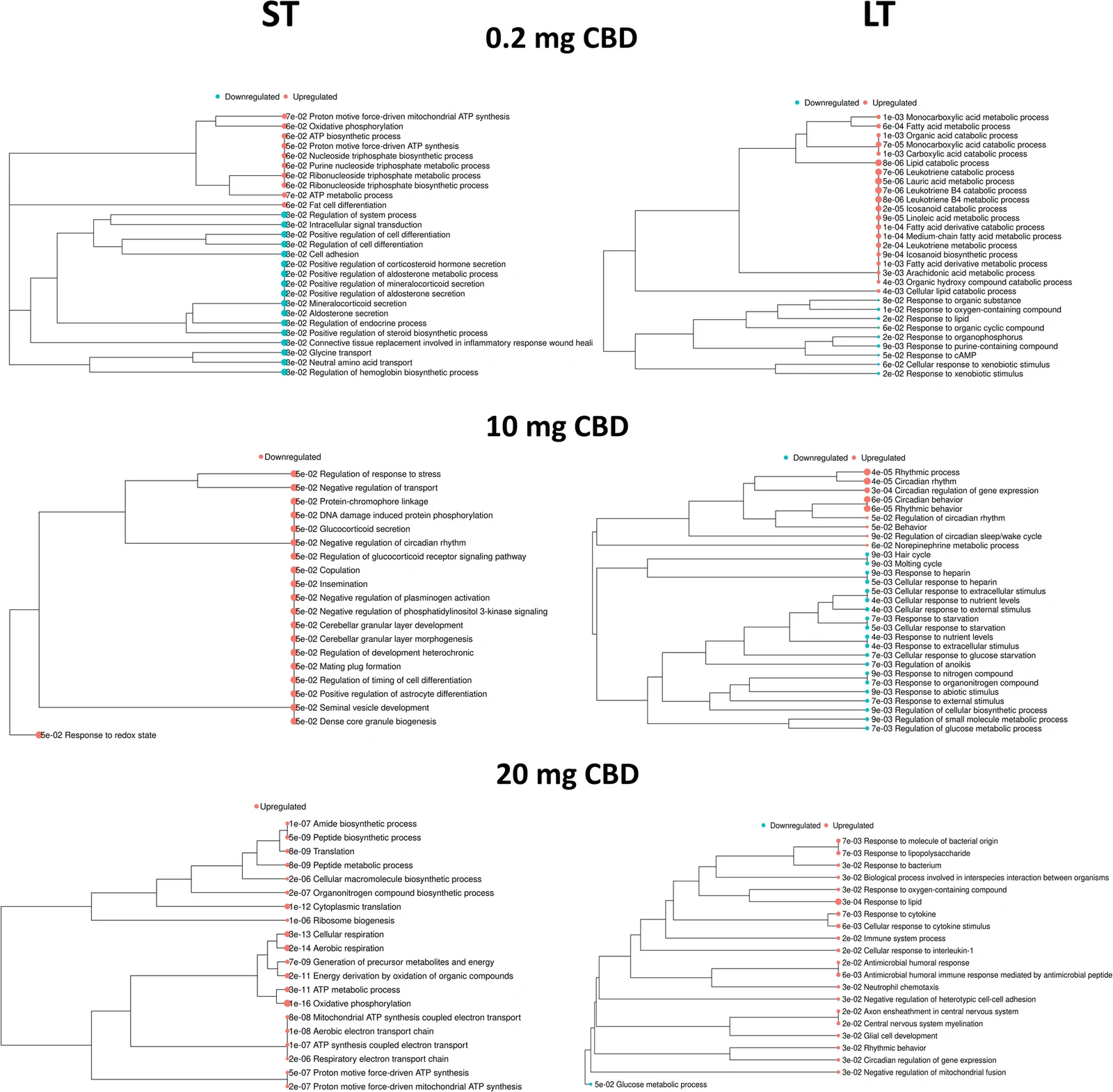
Hierarchical tree of the top 20 biological processes identified by overrepresentation analysis of genes altered by individual CBD treatments in liver. ST – short term, 2 days; LT – long term, 28 days

Genes upregulated by the 10 mg/kg CBD dose in ST treatment were enriched (FDR < 0.05) in major BP categories associated with endocrine and steroid hormone regulation, with a particular emphasis on glucocorticoid and corticosteroid secretion and receptor signaling; stress and DNA damage response pathways, including DNA damage–induced phosphorylation, redox responses, and regulation of damage signaling; neurodevelopmental processes, notably cerebellar development and astrocyte differentiation; and reproductive processes, such as copulation, insemination, mating plug formation, and seminal vesicle development. In addition, a recurrent theme of negative regulation of signaling, transport, and hemostatic processes was observed, including PI3K signaling, platelet aggregation, and general transport mechanisms. Downregulated genes at this dose did not show significant overrepresentation of BP categories (Supplementary File 4, Fig. 3).

For the 20 mg/kg CBD dose, only upregulated genes in ST treatment showed significant BP overrepresentation. These were predominantly associated with mitochondrial energy metabolism and oxidative phosphorylation (including aerobic respiration, electron transport chain activity, and ATP synthesis), protein synthesis and ribosome biogenesis (covering cytoplasmic translation, rRNA processing, ribosomal subunit assembly, and macromolecule biosynthesis), and nucleotide and nitrogen compound metabolism (notably purine nucleoside and ribonucleotide biosynthesis and ATP metabolic processes). Additionally, a smaller but consistent enrichment was observed for humoral innate immune responses, particularly antimicrobial peptide–mediated defense (Supplementary File 4, Fig. 3).

In case of LT treatment, the upregulated genes affected by the lowest applied CBD dose enriched (FDR < 0.05) groups of biological processes involving lipid and fatty acid metabolism, including catabolic and biosynthetic pathways of fatty acids, glycerolipids, and lipid derivatives, with a strong emphasis on eicosanoid, leukotriene, and arachidonic/linoleic acid metabolism. These were accompanied by broader organic acid and small-molecule catabolic processes, as well as regulatory pathways controlling triglyceride and lipid homeostasis. A secondary but distinct group comprises developmental and differentiation-related processes, including renal, urogenital, and multicellular organism development, alongside nervous system–associated processes such as axonal protein localisation and regulation of nerve impulse transmission. In the case of downregulated genes, they were enriched in major categories, including a coordinated cellular response to diverse chemical stimuli, encompassing purine-containing and oxygen-containing compounds, xenobiotics, lipids, and organophosphorus substances (Supplementary File 4, Fig. 3).

For LT treatment with 20 mg/kg CBD, the upregulated genes were overrepresented (FDR < 0.05) in major classes of BP, including circadian and rhythmic biological processes, highlighting regulation of the molecular clock, circadian control of gene expression, and rhythmic behaviors. The downregulated genes enriched terms delineate several major biological process groups, including cellular and organismal responses to extracellular, nutritional, and metabolic stimuli (notably starvation, glucose availability, lipids, amino acids, and nitrogen-containing compounds), coupled with regulation of carbohydrate, small-molecule, and overall metabolic processes. Prominent regulatory layers involved transcriptional repression, modulation of biosynthetic activity, protein phosphorylation and modification, and attenuation of glucocorticoid receptor signalling. Additional process clusters point to stress and homeostasis mechanisms (redox response, anoikis), circadian regulation, and broader developmental programs, encompassing multicellular organism development and specific morphogenetic events (Supplementary File 4, Fig. 3).

The highest applied CBD dose upregulated genes that predominantly enriched (FDR < 0.05) processes such as activation of innate immune and inflammatory responses, including antimicrobial humoral immunity, cytokine- and interleukin-1–mediated signaling, leukocyte and neutrophil chemotaxis, and cellular responses to bacterial components such as lipopolysaccharide. These immune-related pathways were accompanied by processes involved in interspecies interactions and regulation of apoptotic and cell–cell adhesion signalling. The downregulated genes were overexpressed in a single BP concerning glucose metabolic process (Supplementary File 4, Fig. 3).

### Changes in the transcriptome of the spleen following CBD treatment

For spleen samples, sequencing generated an average of 11.3 million (SD = 2.8 million) 3′ mRNA-Seq reads per sample. On average, 98.6% of reads passed initial quality filtering. Of the filtered reads, a mean of 65.2% (SD = 13.5) were uniquely mapped to the reference genome. The majority of these reads were assigned to annotated genes (65.8%), corresponding to an average of 5.9 million reads per sample (Supplementary File 2).

**Fig. 4:**
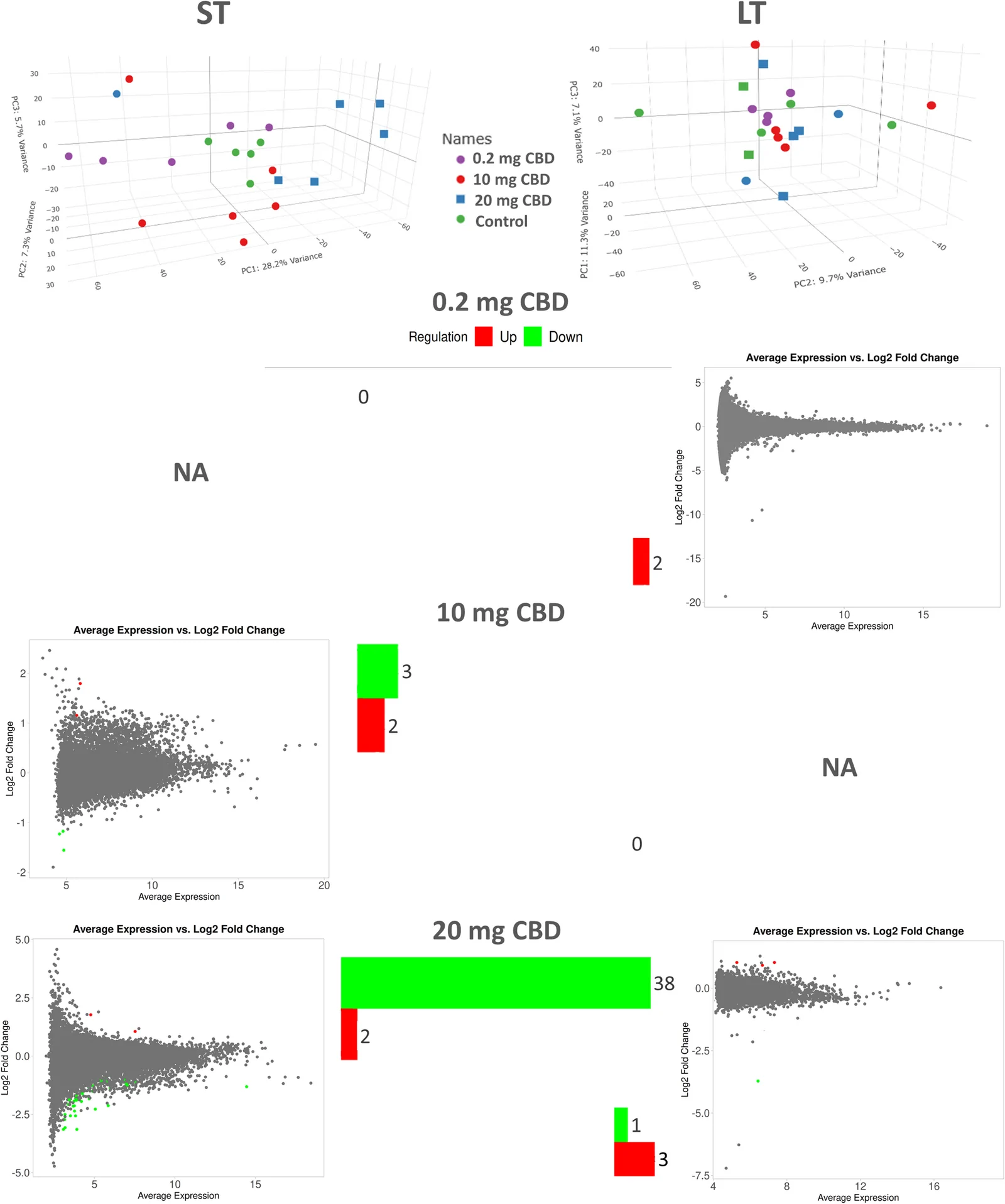
Changes in the splenic transcriptional profile following CBD administration for two (ST) or 28 (LT) days. The figure presents PCA results for the first three principal components and MA plots illustrating gene expression changes induced by individual CBD doses. The number of differentially expressed genes identified for each treatment is also shown

Initial evaluation of spleen transcriptomic profiles in the ST treatment group using principal component analysis (PCA) revealed that two control samples and one sample from the 0.2 mg/kg CBD-treated group exhibited either low read counts or strong deviation of expression profile from the remaining samples within their respective groups. These samples were therefore excluded from further analyses. The remaining samples showed partial overlap between groups, with the greatest divergence in expression profiles observed in animals treated with the highest CBD dose (Fig. 4).

In the LT treatment group, PCA revealed substantial overlap among all study groups, with no clear separation of expression profiles with respect to CBD treatments. Some within-group heterogeneity was observed, with the highest variance detected in the control group and in animals treated with 10 mg/kg b.w. CBD (Fig. 4).

The applied CBD treatment in the ST group resulted in differential expression of a relatively small number of genes. The lowest CBD dose did not induce any significant changes in gene expression, whereas the intermediate dose altered the expression of five genes (two upregulated and one downregulated), and the highest CBD dose affected 40 genes, of which 38 were downregulated.

In the LT treatment group, the number of differentially expressed genes was even lower. The lowest CBD dose led to the upregulation of two genes, while the highest dose altered the expression of four genes (three upregulated and one downregulated) (Fig. 4; Supplementary File 5). In the ST treatment, *Cadm2* gene was commonly affected by two different CBD doses, whereas in the long-term (LT) treatment, the only gene shared between the two treatment groups was *Fkbp5*. No genes were commonly altered between the ST and LT treatments (Supplementary File 5).

**Fig. 5:**
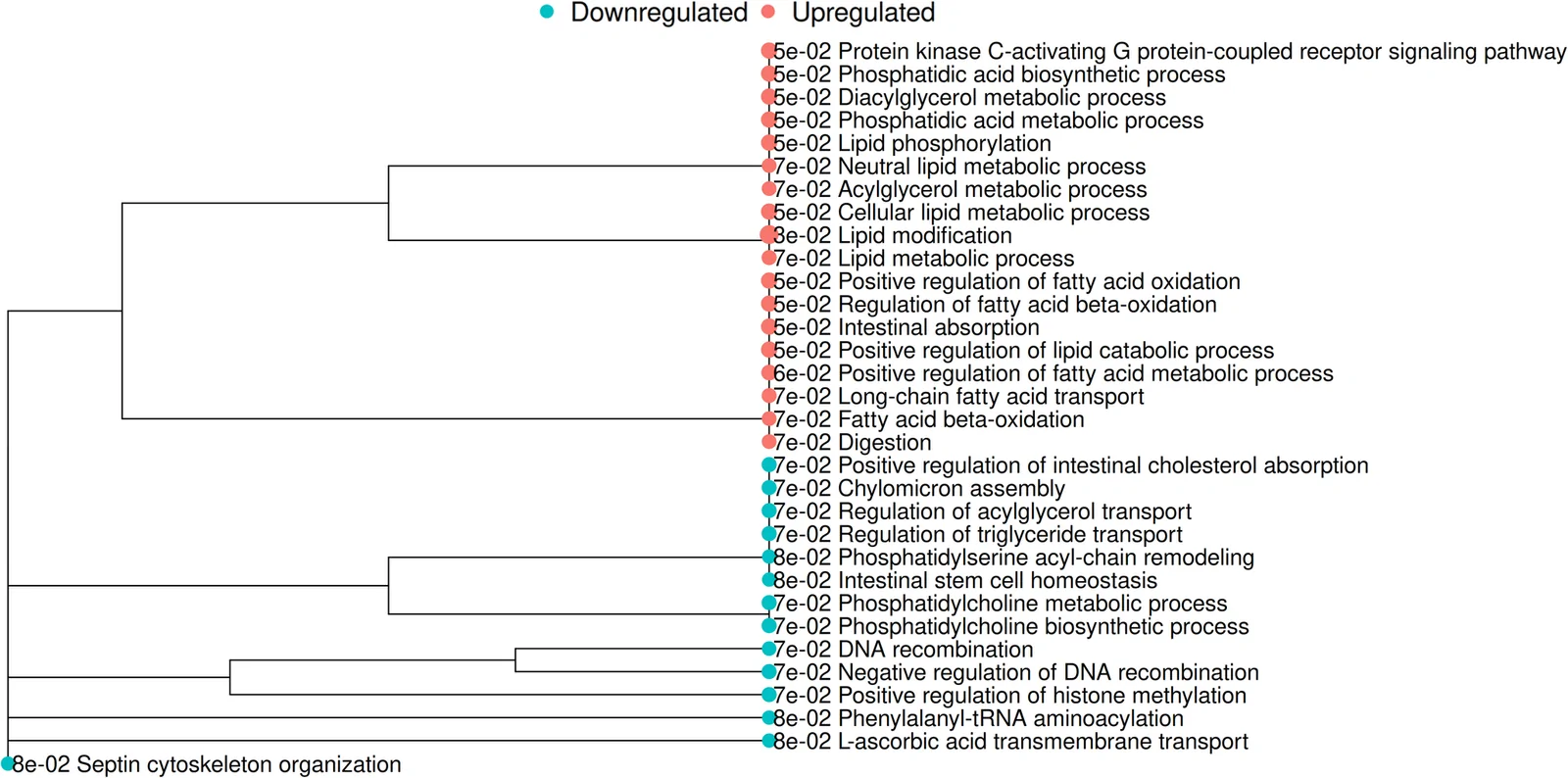
Hierarchical tree of the top 20 biological processes identified by overrepresentation analysis of genes altered in the spleen following short-term (ST) CBD treatment (20 mg/kg). ST – short term, 2 days; LT – long term, 28 days

In the spleen transcriptome analysis, overrepresentation tests were conducted only for comparisons with a higher number of differentially expressed genes, specifically the 40 genes altered by the 20 mg/kg CBD dose in the ST treatment. This analysis identified a single process enriched for upregulated genes (FDR < 0.05) related to lipid modification. However, several other processes associated with lipid metabolism and the regulation of lipid-related pathways showed a statistical trend toward enrichment (Fig. 5; Supplementary File 6).

### Results of qPCR validation on RNA-Seq

The results of the qPCR validation of the RNA-Seq data showed good concordance, with moderate-to-high correlation coefficients for most genes (*r* = 0.69–0.90). However, one gene (*Ighg2b*) exhibited a low correlation coefficient (*r* = 0.316). All of the highest correlation coefficients were statistically significant, with p-values ranging from 0.012 to 1.2 × 10⁻⁷ (Supplementary File 7).

## Discussion

The present study shows that CBD exerts coordinated systemic and organ-specific immunomodulatory effects, characterized by reduced circulating pro-inflammatory cytokines, pronounced dose- and time-dependent transcriptional remodeling in the liver, and comparatively modest changes in the spleen. Importantly, the integration of cytokine and transcriptomic data indicates that these effects are not independent but reflect interconnected regulation across systemic and tissue -specific levels. Altogether, these findings suggest that CBD may function not only as an anti-inflammatory agent but as a modulator of immunometabolic networks.

### Effects of CBD on systemic pro-inflammatory cytokines

In the present study, CBD administration led to significant reductions in IL-1β and TNF-α concentrations in the ST treatment group. In contrast, IL-6 changes were primarily observed during LT exposure, with the strongest effect detected at mid-treatment and partial persistence at the end of treatment. TNF-α was also reduced after LT exposure at day 28, suggesting sustained suppression of specific pro-inflammatory outputs despite the delayed IL-6 pattern. Notably, these temporal patterns in cytokine modulation correspond with the observed organ-specific transcriptomic responses, particularly the pronounced hepatic remodeling and comparatively limited splenic changes, suggesting that systemic cytokine regulation may be linked to tissue-specific molecular adaptations rather than uniform immune suppression.

These results are consistent with the studies of the other authors in which CBD has demonstrated anti-inflammatory properties, in particular in the case of lowering cytokine production in both central and peripheral immune models. In the results of Kozela et al. (2015) and Atalay et al. (2020) CBD was shown to be an inhibitor of NF-κB, which is a known central transcription factor regulating TNF-α and IL-1β expression. Moreover, CBD indicated the suppression of the expression of pro inflammatory mediators (including IL-1β and TNF-α) and inducible nitric oxide synthase (iNOS) (Esposito et al. 2007; Kozela et al. 2015). In addition, various studies have shown that cannabinoids can regulate cytokine production through mechanisms involving CB2 receptor signaling, both through CB2 dependent and CB2 independent mechanisms (Klein 2005; Pertwee 2008).

The regulation of IL-6 is particularly interesting in the concept of CBD administration. IL-6 is a pleiotropic cytokine that plays a central role in acute-phase responses, B-cell differentiation, and the maintenance of chronic inflammatory signaling (Tanaka et al. 2014). In our study, the reduction in IL-6 levels is especially visible during the prolonged treatment, which strongly suggests that this effect may results from some other regulatory mechanisms than a direct transcriptional suppression. Given that IL-6 production is frequently induced downstream of IL-1β and TNF-α signaling cascades, its delayed decline may reflect a gradual attenuation of upstream inflammatory stimuli. This delayed cytokine response is consistent with the transcriptomic findings, where long-term CBD treatment was associated with enrichment of metabolic and circadian pathways rather than immediate immune signaling changes, supporting the idea that IL-6 modulation may arise from secondary, system-level regulatory processes. It is important to notice that CBD is not a traditional agonist of the CB1 or CB2 receptors. Instead, it has a low affinity for canonical cannabinoid receptors and modulates a variety of molecular targets, such as redox-sensitive pathways, transient receptor potential channels (TRPV1), peroxisome proliferator-activated receptor gamma (PPARγ), and adenosine signaling (O’Sullivan 2007; Pertwee 2008; Atalay et al. 2020). This multimodal mechanism likely explains the gradual and dose-dependent cytokine modulation observed here.

### Liver transcriptomic changes linking metabolism and immune regulation

An essential organ for metabolism but also immunity in the body, the liver combines metabolic homeostasis with systemic inflammatory signals (Robinson et al. 2016). Significant hepatic pathway enrichment patterns that differed by dosage and treatment duration were found in our transcriptome data.

Short-term high-dose CBD exposure was related to the enrichment of altered genes in the oxidative phosphorylation and ribosomal biogenesis pathways. Cannabidiol has been reported to affect mitochondrial function and bioenergetics, including the modulation of calcium flux, mitochondrial membrane potential, and reactive oxygen species (ROS) production (Atalay et al. 2020; Chan and Duncan 2021). Mitochondrial metabolism is closely connected to immune cell activation and inflammatory responses, as shifts between oxidative phosphorylation and glycolysis can shape cytokine production and influence immune cell polarization. Thus, the observed upregulation in mitochondrial pathways may be a sign of adaptive metabolic reprogramming instead of toxicity or hyperactivation. Importantly, these hepatic transcriptional changes coincide with the early reduction in circulating IL-1β and TNF-α, suggesting that metabolic reprogramming in the liver may contribute to the modulation of systemic inflammatory outputs. The interplay between immune signaling and cellular metabolism is increasingly recognized as a fundamental regulator of inflammatory responses, although this concept is not specific to CBD and represents a well-established principle in immunometabolism (Zhang et al. 2018; Trinchese et al. 2024).

Long-term CBD administration resulted in alterations of genes that were enriched in fatty acid metabolism and arachidonic acid–related pathways. This finding is relevant because lipid mediators derived from arachidonic acid (eicosanoids) play pivotal roles in inflammation responses. Cannabinoids affect endocannabinoid and lipid signaling networks, in part by altering fatty acid amide hydrolase (FAAH) and cyclooxygenase pathways (Pertwee 2008). Additionally, the activation of PPARγ by CBD has been observed which is supported by the O’Sullivan study (2007)(O’Sullivan 2007). PPARγ controls lipid metabolism and contributes to anti-inflammatory responses by trans-repression of NF-κB and AP-1 signaling pathways (Ricote and Glass 2007). The combination of lipid metabolic pathway enrichment with systemic cytokine reduction indicates that CBD may promote metabolic conditions that contribute to the regulation of inflammatory responses rather than simply inhibiting them. This supports a model in which hepatic lipid signaling pathways indirectly influence cytokine production, linking organ-specific metabolic changes with systemic immune modulation.

A potential interaction between CBD and endocrine regulatory systems is suggested by the enrichment of altered genes in pathways related to glucocorticoids and steroids at particular dosages. Glucocorticoids are well-known immunomodulators that lower leukocyte activation and block the transcription of pro-inflammatory cytokines (Coutinho and Chapman 2011; Strehl et al. 2019). Although CBD does not act as a glucocorticoid receptor agonist, interactions between cannabinoid signaling and stress hormone pathways have been described (Pertwee 2008). Indirect integration between endocrine and immune regulatory mechanisms may therefore be indicated by the altered expression of steroid metabolism genes in the liver.

In addition, one of the most interesting results was the enrichment of circadian rhythm-associated genes and pathways, including differential regulation of *Nr1d1* (REV-ERBα) gene expression. The nuclear receptor REV-ERBα controls the expression of genes involved in inflammation and metabolism and is a fundamental part of the molecular circadian clock. In the research of (Gibbs et al. 2012) it was shown that REV-ERBα directly modulates innate immune responses and cytokine production, linking circadian oscillations to the regulation of inflammation. Circadian regulation of immune function is now well established, with leukocyte trafficking, cytokine secretion, and inflammatory responses displaying robust diurnal variation (Scheiermann et al. 2013). Notably, the highest CBD dose was also associated with enrichment of gene sets related to innate immune–associated signaling (including interleukin-1–related pathways and leukocyte chemotaxis), indicating a dose-dependent shift in hepatic transcriptional signatures rather than overt inflammatory activation. Despite the enrichment of immune-related pathways at the transcriptional level, circulating cytokine levels remained reduced, further supporting the notion that transcriptional activation does not necessarily translate into increased systemic inflammation but may reflect regulatory or adaptive processes. Interestingly, in our previous work, transcriptomic changes in kidney tissues following LT CB treatment showed similar groups of affected processes, including the regulation of genes related to circadian rhythm (e.g., *Nr1d1*, also affected in liver), glucocorticoid receptor function (e.g., *Cyp1b1*,* Ddit4*,* Foxo3*,* Gjb2*), lipid metabolism (e.g., *Cyp2d22*,* Cyp2d9*,* Decr2 Hacl1*), and inflammatory response (e.g., *Cxcr4*,* Ccl28*) (Rokicki et al. 2024).

### Spleen transcriptomic changes in immune regulation

The spleen is a vital mediator of systemic cytokine production and a significant repository of immune cells. Immune tissues are known to have high expression levels of cannabinoid receptors, in particular CB2 receptors (Klein 2005). Consequently, splenic leukocyte activation states may change due to CBD’s indirect modulation of CB2 signaling and other immune pathways (Turkki et al. 2026). However, in our dataset, the spleen exhibited relatively modest transcriptional changes compared with the liver, particularly in the long-term treatment group, where few differentially expressed genes were detected. This limited splenic response, in contrast to the pronounced hepatic remodeling, suggests that systemic cytokine changes are not primarily driven by classical immune organs but may instead reflect indirect regulation mediated by metabolically active tissues such as the liver. The most evident spleen response was observed after short-term exposure to the highest CBD dose, where the altered genes showed signals related mainly to lipid-associated processes. Overall, the combined cytokine and transcriptomic patterns, particularly the robust hepatic response, suggest that CBD may modulate immune signaling rather than uniformly suppress it. This modulation could reflect changes in immune cell populations, activation status, or gene expression patterns without promoting excessive inflammatory cytokine release. This explanation is consistent with earlier findings that cannabinoids have immunological effects that are context-dependent and occasionally biphasic (Klein 2005; Pertwee 2008). Given the spleen’s central role in coordinating innate and adaptive immune responses (Lewis et al. 2019), splenic transcriptional changes could be related to systemic cytokine modulation; however, in our dataset the spleen showed only limited differential expression, particularly after long-term exposure. These findings further support the notion that CBD-mediated immune modulation involves coordinated adjustments in immune cell activation and signaling rather than simple suppression of inflammatory output. This non-linear pharmacological profile is supported by dose-dependent divergence in transcriptomic signatures observed in this study.

Cannabinoids frequently exhibit nonlinear dose-dependent effects. According to Campos et al. (2012), CBD interacts with several signaling pathways, and depending on the dosage, these pathways’ relative contributions may change. While higher doses can activate compensatory regulatory mechanisms or engage additional molecular targets, lower doses may result in the selective modulation of specific pathways. Our results support this concept. While extended treatment was linked primarily to circadian-related gene expression changes, the highest CBD dose was associated with enrichment of innate immune–associated signaling. Despite these transcriptional differences, systemic levels of pro-inflammatory cytokines remained reduced, indicating that changes in gene expression do not necessarily correspond to increased inflammatory activity. These findings imply that CBD may encourage adaptive modifications within immune–metabolic networks. From a therapeutic perspective, this emphasizes how crucial it is to carefully consider dosage and length of treatment when assessing CBD’s potential future clinical effects. Importantly, the integration of systemic cytokine data with organ-specific transcriptomic profiles suggests coordinated multi-organ regulation. Rather than representing independent effects, the observed cytokine and transcriptomic changes appear to form a coherent response, in which hepatic metabolic and regulatory pathways contribute to the shaping of systemic immune outputs.

### CBD immunomodulatory mechanisms in current literature

Comprehensive reviews confirm that CBD demonstrates anti-inflammatory, antioxidant, and immunoregulatory properties via various mechanisms, including NF-κB inhibition, ROS modulation, adenosine uptake inhibition, and PPARγ activation (Iffland and Grotenhermen 2017; Atalay et al. 2020). Mujahid et al. (2025) reviewed the immunomodulatory properties of CBD, highlighting preclinical and clinical evidence supporting its potential efficacy in Type 1 diabetes, an immunometabolic disease. Similarly, (Peltner et al. 2023), using human cells and cell-free assays, demonstrated that CBD exerts anti-inflammatory effects by inducing a switch in lipid mediator production, suggesting that CBD may modulate immune–metabolic pathways. More recently, Rueda-Munguía et al. (2026) showed in human macrophages that CBD selectively attenuates lipotoxic immunometabolic inflammation, further supporting a role for CBD in the regulation of the interplay between inflammatory and metabolic processes. Our results enhance this framework by demonstrating synchronized organ-specific transcriptomic remodeling, especially concerning genes of circadian and metabolic regulators. The convergence of mitochondrial, lipid, circadian, and cytokine pathways in our gene enrichment analysis indicates that CBD presumably affects essential regulatory centers rather than specific inflammatory mediators. This systems-level modulation may be the reason for the reported health benefits in inflammatory, autoimmune, and metabolic disorders.

### Temporal differences between short-term and long-term CBD responses

One of the most remarkable observations of this study is the clear divergence between the effects of short-term and long-term CBD administration. Short-term treatment was characterized by fast suppression of circulating IL-1β and TNF-α accompanied by clear hepatic transcriptional changes involving mitochondrial function, oxidative phosphorylation, ribosome biogenesis, and glucocorticoid-related pathways. These observations suggest that the initial response to CBD primarily involves acute metabolic adaptation accompanied by early regulation of inflammatory mediators, consistent with the close interplay between mitochondrial metabolism and immune function (O’Neill et al. 2016; Trinchese et al. 2024). In contrast, prolonged CBD exposure resulted in a distinct transcriptional profile dominated by lipid and fatty acid metabolism, circadian rhythm regulation, and broader immunometabolic pathways, while systemic cytokine modulation shifted toward sustained suppression of IL-6 and persistent reduction of TNF-α. This temporal transition from early bioenergetic and stress-related responses to longer-term metabolic reprogramming suggests that CBD does not elicit a static biological response but rather induces progressive physiological adaptation. Such findings are consistent with the pharmacokinetic properties of CBD, which, owing to its high lipophilicity, is widely distributed into highly perfused and lipid-rich tissues, including the liver and adipose tissue, following repeated administration (Taylor et al. 2018; Martinez Naya et al. 2024). This prolonged tissue exposure may alter the relative contribution of different molecular pathways over time rather than simply intensifying the initial pharmacological response, reflecting the multimodal mechanism of action of CBD (Campos et al. 2012; Martinez Naya et al. 2023). Consequently, the biological effects of CBD appear to depend not only on dose but also on treatment duration, emphasizing the importance of exposure time when interpreting both experimental findings and the potential therapeutic actions of this phytocannabinoid.

## Limitations and future directions

The present study has several limitations that should be acknowledged. Although transcriptomic profiling provides a comprehensive and unbiased overview of CBD-induced molecular alterations, the identified pathways were not validated by targeted functional assays. Therefore, the proposed biological mechanisms should be considered hypothesis-generating and require confirmation in future studies. Second, the use of bulk RNA sequencing does not allow discrimination between changes in gene expression within individual cell types and shifts in tissue cellular composition. Complementary approaches, such as flow cytometry, single-cell transcriptomics, or targeted biochemical analyses, would provide additional mechanistic insight into the immunometabolic effects of CBD. What is more, although Tween-80 (used as vehicle) and isoflurane (used for anesthesia in the euthanasia process) have been reported to exhibit immunomodulatory properties, these agents were administered equally to all experimental groups, including the controls, thereby minimising their potential impact on the interpretation of CBD-specific effects.

An additional limitation of this study is the inclusion of only male mice in the experiment. As sex-dependent differences in endocannabinoid signaling, immune responses, and metabolic regulation have been widely reported, the findings presented here may not be fully generalizable to females. Future investigations should therefore include both male and female animals to evaluate potential sex-specific responses to CBD treatment.

Furthermore, Additional research into the temporal dynamics between circadian gene modulation and cytokine suppression would also be valuable, especially considering the emerging function of REV-ERBα in inflammatory regulation.

## Conclusions

In this study, we show that CBD administration in mice reduces systemic pro-inflammatory cytokines while inducing dose- and time-dependent transcriptional remodeling in the liver, with comparatively modest transcriptomic changes in the spleen. These changes involve genes related to mitochondrial bioenergetics, lipid metabolism, glucocorticoid-associated pathways, and include circadian regulators such as REV-ERBα-coding genes. CBD does not seem to work as a general immunosuppressant; instead, it seems to work as a complex immunometabolic modulator that helps keep the immune system in balance by coordinating transcriptional reprogramming.

## Supplementary Information

Below is the link to the electronic supplementary material.

Raw results of cytokine concentrations in the blood plasma of control and CBD-treated mice

Read counts and mapping statistics for liver and spleen RNA-Seq data

Differential expression and comparative analyses of genes following CBD treatment in the liver

Results of overrepresentation analysis of GO biological process categories for genes altered by CBD treatment in the liver

Differential expression and comparative analyses of genes following CBD treatment in the spleen

Results of overrepresentation analysis of GO biological process categories for genes altered by ST 20 mg/kg CBD treatment in the spleen

Primers and results of qPCR validation of RNA-Seq

## Data Availability

Raw reads, as well as raw read counts, were deposited in the Gene Expression Omnibus (GEO) and Sequence Read Archive (SRA) databases from the National Center for Biotechnology Information (NCBI) under the accession numbers: GEO - GSE261716 and SRA BioProject PRJNA1088484 for liver; GEO - GSE328789 and SRA BioProject PRJNA1456538 for spleen.
